# Generating minimum set of gRNA to cover multiple targets in multiple genomes with MINORg

**DOI:** 10.1093/nar/gkad142

**Published:** 2023-03-15

**Authors:** Rachelle R Q Lee, Wei Yuan Cher, Jinge Wang, Yujie Chen, Eunyoung Chae

**Affiliations:** Department of Biological Sciences, National University of Singapore, Singapore 117558, Singapore; Department of Biological Sciences, National University of Singapore, Singapore 117558, Singapore; Department of Biological Sciences, National University of Singapore, Singapore 117558, Singapore; Department of Biological Sciences, National University of Singapore, Singapore 117558, Singapore; School of Life Science and Technology, Xi'an Jiaotong University, Xi'an, Shaanxi 710049, P. R. China; Department of Biological Sciences, National University of Singapore, Singapore 117558, Singapore

## Abstract

MINORg is an offline gRNA design tool that generates the smallest possible combination of gRNA capable of covering all desired targets in multiple non-reference genomes. As interest in pangenomic research grows, so does the workload required for large screens in multiple individuals. MINORg aims to lessen this workload by capitalising on sequence homology to favour multi-target gRNA while simultaneously screening multiple genetic backgrounds in order to generate reusable gRNA panels. We demonstrated the practical application of MINORg by knocking out 11 homologous genes tandemly arrayed in a multi-gene cluster in two *Arabidopsis thaliana* lineages using three gRNA output by MINORg. We also described a new PCR-free modular cloning system for multiplexing gRNA, and used it to knockout three tandemly arrayed genes in another multi-gene cluster with gRNA designed by MINORg. Source code is freely available at https://github.com/rlrq/MINORg.

## INTRODUCTION

In functional genomics, gene function is frequently investigated using knockdown or knockout techniques and observing any changes to phenotype. The clustered regularly interspaced short palindromic repeats-Cas (CRISPR-Cas) system (1,2) has come to dominate the field of gene editing. Unlike older gene-editing tools such as zinc-finger nucleases (ZFN) (3) and transcription activator-like effector nucleases (TALEN) (4) that recognise DNA motifs through their protein structures, CRISPR-Cas systems owe their specificity to a short guide RNA (gRNA) sequence that complementary base pairs with a target sequence. Consequently, the CRISPR-Cas system easily lends itself to multiplexing as only the gRNA has to be tailored for each target (5).

A pangenome is the genomic totality of a taxon, comprising the core genome shared by all individuals in a given taxon and dispensable genes which are found in only a subset of individuals (6). Falling costs and increasing availability of whole-genome sequencing have made the study of pangenomes more attractive and widespread (7–10). Thus, it is now possible to investigate the function of genes across various genetic backgrounds rather than a single reference genome. However, intraspecific variation in target and background sequences may alter the ability of a single gRNA to direct a CRISPR-Cas construct to a desired genomic destination as well as the likelihood of off-target effects in non-reference individuals.

Existing gRNA design tools rarely account for intraspecific variation in non-reference genomes, and, where they do, off-target effects are usually only checked against a single genetic background (sometimes together with a reference genome). Furthermore, the experimental burden of designing and cloning separately designed gRNA for multiple genes in multiple genomes may render large pangenomic screens tedious, which highlights the need for gRNA design tools to be able to generate a minimum gRNA set capable of covering all desired targets in the pangenome era. Recent tools such as MultiTargeter (11), which designs minimum gRNA for multiple targets, GuideMaker (12), which designs gRNA in non-reference genomes, and CRISPR-Local (13), which designs minimum gRNA for multiple targets in non-reference genomes on a per-genome basis, address some but not all of these considerations.

Therefore, we have created MINORg to take into account all of these limitations simultaneously and output minimum gRNA sets that cover all desired targets in all desired backgrounds. Additionally, MINORg also allows users to infer homologues in unannotated non-reference genomes and define them as targets, as well as design gRNA in user-specified protein domains or gene features (such as the 5’ untranslated region (UTR)).

## MATERIALS AND METHODS

### MINORg algorithm

**Figure 1.**
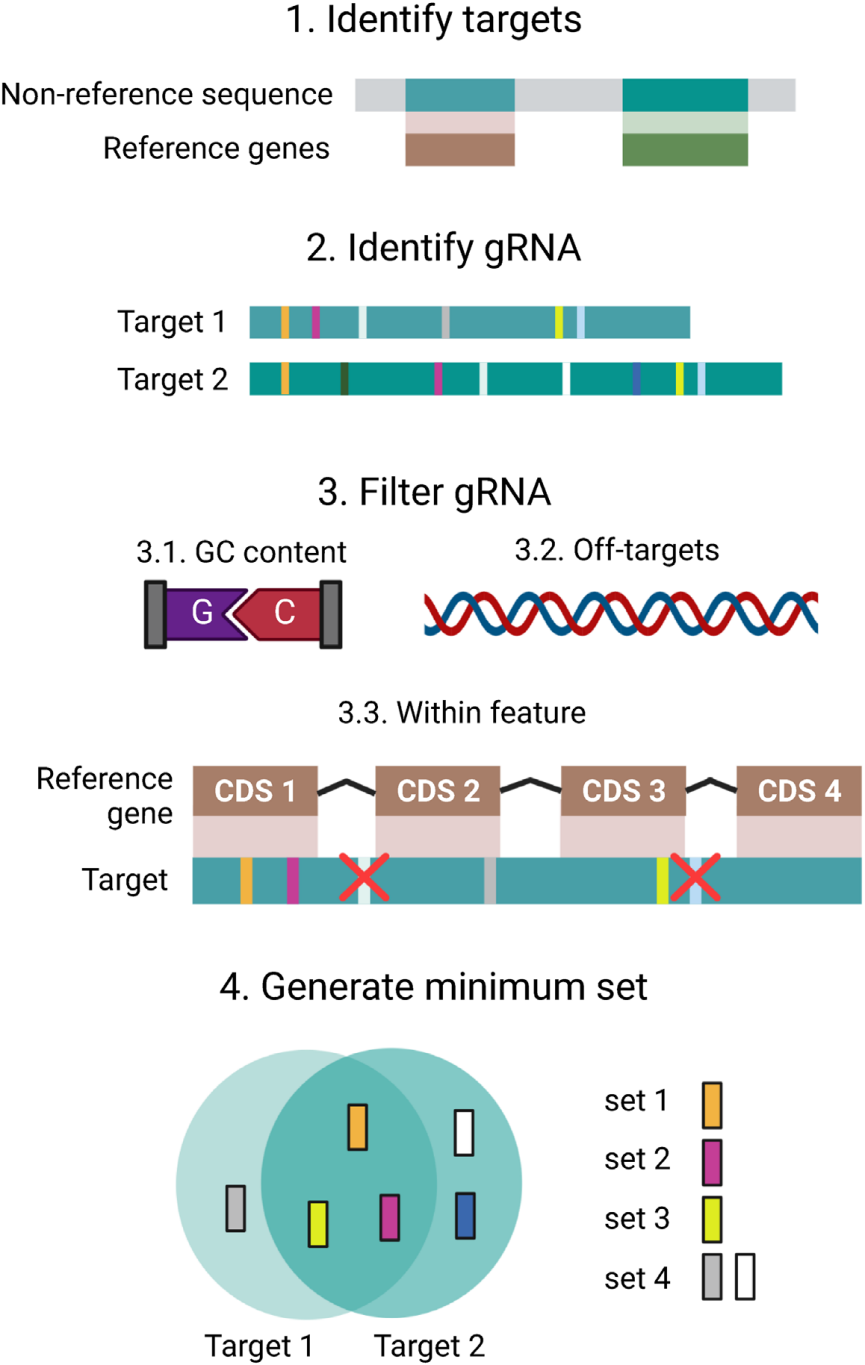
MINORg overview. The full programme consists of four steps. In step 1, gRNA targets are identified by BLASTN of reference genes to non-reference genomes. The targets are represented in green. This step will be skipped if a user directly supplies their desired target sequences, or if only reference genes are targeted. In step 2, gRNA are generated from target sequences identified in step 1. Each unique gRNA sequence is represented with a different colour. In step 3, gRNA are filtered by GC content, off-target effects, as well as whether they are found within a desired feature. If gene annotations have been provided, gRNA are removed if they do not fall within reference gene CDS regions after alignment of targets with reference genes. Finally, in step 4, minimum gRNA sets are generated, with the goal of covering all targets using the least number of gRNA. Created with BioRender.com.

MINORg, which is written using Python3 (14), consists broadly of four different steps: 1. Identification of orthologues of desired genes in non-reference genomes. 2. Generation of all possible gRNA from sequences output by step 1. 3. Filtering of candidate gRNA for on-target and off-target specificity. 4. Generation of a minimum set of gRNA that can target sequences output by step 1 (Figure 1). Each of the four steps can also be executed independently to facilitate parameter optimisation as well as integration of results from other on- and off-target assessment tools.

The first step of orthologue identification is based on local BLAST (15,16). It executes BLASTN locally using reference genes as query and non-reference genomes as subject, merges hits within a certain allowable distance, and filters for minimum length and percentage identity to reference genes. All these parameters can be tuned by the user based on the rate of polymorphisms of their set of genes. Users may additionally restrict the search to a specific protein domain using a Reverse Position-Specific BLAST (RPS-BLAST) database and specifying the domain’s position-specific scoring matrix (PSSM) ID. The output of this step is a set of sequences that the tool will attempt to generate gRNA for. Users who already have the sequences they intend to target may skip this discovery step.

The second step is the most straightforward. Based on a user-provided PAM pattern and gRNA length, all possible gRNA will be generated from all sequences output by step 1. We have implemented a flexible method of defining PAM. It allows for upstream PAM, spacer length not equal to one, ambiguous bases, PAM-less, and/or multi-PAM gRNA identification. This implementation uses a stripped-down version of regular expressions. We believe it is important to make a gRNA tool agnostic to any CRISPR-Cas system to both cater to a variety of systems available now and also to future-proof MINORg against future development of novel CRISPR-Cas technologies.

The third step employs three main gRNA filters: (i) GC content, (ii) off-target effects, (iii) within feature. GC content filtering is straightforward, with default minimum and maximum GC content set at 0.3 and 0.7, although both are user-adjustable. Off-target effects are assessed by the presence of gRNA sequences outside of target regions.

Unlike gRNA off-target assessment in currently available tools, Primer-BLAST (17) will be employed to search for such regions for each gRNA in both the reference genome and the non-reference genome provided to the tool for orthologue discovery in step 1. The user may also provide a custom set of sequences to be screened against. By default, MINORg filters off-target hits by comparing the number of gaps, mismatches, and/or unaligned positions across an entire gRNA against user-specified thresholds. However, as mismatch tolerance across a gRNA generally increases with distance from PAM site, MINORg also allows users to customise different thresholds for different positions along a gRNA. The default behaviour is to exclude only gRNA with perfect alignment elsewhere in a non-target region. Users may adjust the thresholds to increase filtering strictness.

Thirdly, gRNA will be filtered for their presence within desired features, such as CDS and 5’ UTR. For non-reference targets that were discovered by the first step in unannotated genomes, we infer the ranges of desired features from alignments with reference genes using MAFFT (18) and retain only gRNA that can target at least one such non-reference sequence in a region that aligns with at least one reference gene’s desired region. This step outputs a mapping file that maps gRNA to their location on targets and tracks the pass/fail status of these filters.

Finally, the fourth step employs one of two set cover algorithms to identify one (or however many requested by the user) minimum gRNA set required to target all sequences output by step 1. Depending on whether the user wishes to prioritise proximity to the 5’ end or non-redundancy, List and Remove (19) or an adapted version of an algorithm to enumerate minimal weight set covers (20) will be executed respectively. We adapted Ajami & Cohen (20) by seeding the algorithm with each gRNA for a fixed number of iterations and using the output to identify reasonable thresholds, following which these thresholds were used to set cutoffs for a brute force search for optimal minimum gRNA sets. This step produces the best results when targets share sequence homology. For gRNA with equivalent coverage (that is, gRNA that are theoretically capable of editing the same combination of targets), the gRNA that is closest to the 5′ end of a target sequence will be prioritised.

Software and algorithms used in MINORg are listed in Table 1.

**Table 1.** Software and algorithms used in MINORg

Resource	Source	Identifier
Python 3	(14)	
Biopython	(21)	
pyfaidx	(22)	
Typer	https://github.com/tiangolo/typer	
Pybedtools	(23)	RRID:SCR_021018
BLAST+	(16)	
BEDTools	(24)	RRID:SCR_006646
MAFFT	(18)	RRID:SCR_011811
List and remove (LAR)	(19)	
Enumerating minimum weight set covers	(20)	

### Modularity and integration of results from other tools

MINORg is designed so that each step can be executed independently. This allows users to incorporate gRNA filtering results from other software to supplement MINORg’s three built-in filters if desired. MINORg generates tab-separated mapping files that map gRNA to their target sequences as well as track filtering status for each gRNA-target pair. To integrate the results of other on- and/or off-target assessment software, users may append additional columns containing pass/fail status of each gRNA-target pair as determined by their on- and/or off-target filtering software to the mapping file. The updated mapping file can then be used as input for a subcommand that executes the last step, which will re-generate minimum gRNA sets from the modified mapping file.

### Design of gRNA for CRISPR-Cas9 knock-out of *RPW8/HR4* genes

We selected two non-reference *Arabidopsis thaliana* lineages (also known as accessions), TueWa1-2 (CS10002) and KZ10 (CS22442), as a testbed for the capability of MINORg to design gRNAs for (1) reference gene homologues and (ii) genes absent in reference genome (iii) across non-reference genomes (iv with a minimum number of gRNAs to target an entire cluster of genes. With MINORg, we designed minimum sets of gRNAs targeting *RPW8/HR4* genes in the two accessions (25) after obtaining cluster sequence and annotations from NCBI’s Nucleotide database (accessions MK598747.1 (TueWa1-2) and KJ634211.1 (KZ10)). The following command was used to run MINORg:


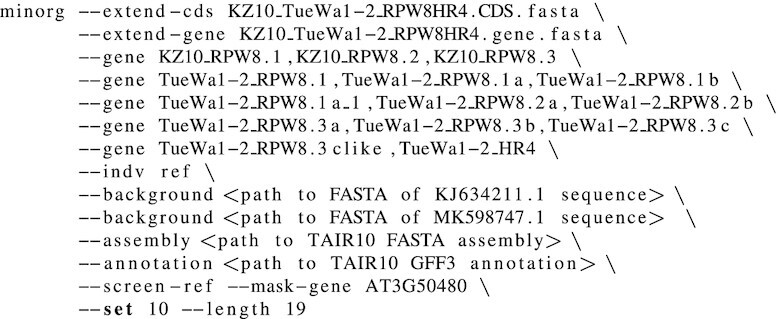


As there are no GFF3 annotations for the cluster for either KZ10 or TueWa1-2, we used ‘- -extend-cds’ and ‘- -extend-gene’ to temporarily add the cluster genes of both accessions to the reference assembly and annotation. These files were manually curated from MK598747.1 and KJ634211.1 sequences and annotations and can be found at https://github.com/rlrq/MINORg/publication_data. Using ‘- -gene’, we then specified our target genes, and ‘- -indv’ specifies that the genes are in the reference genome. The genomic sequences for the full *RPW8/HR* clusters (including paralogous *HR1/2/3* which were not included in our target genes) were supplied for off-target screening using ‘- -background’. ‘- -assembly’ and ‘- -annotation’ together specify the reference *A. thaliana* genome (TAIR10; retrieved from https://www.arabidopsis.org/download/index-auto.jsp?dir=%2Fdownload_files%2FGenes%2FTAIR10_genome_release (annotation)), while ‘- -screen-ref’ informs MINORg to also screen the reference genome for off-targets. ‘- -mask-gene’ hides *HR4* (gene ID AT3G50480) in the reference genome from the off-target filter as its orthologues in TueWa1-2 and KZ10 are target genes. Finally, ‘- -length’ specifies gRNA length, and ‘- -set’ determines how many mutually exclusive gRNA sets to generate. All other parameters (including 3’ NGG PAM, restricting gRNA to CDS regions, 30% ≤ GC ≤ 70%, and 0 mismatch off-target threshold) were left as default.

We selected two sets of gRNA output by MINORg (Supplementary Table S1) for further experiments.

### Design of gRNA for CRISPR-Cas9 knock-out of *NRG1* genes

To demonstrate multi-gene targeting using a multiplexed construct, we designed gRNA for three *N REQUIREMENT GENE 1* (*NRG1*) paralogues: *NRG1.1* (gene ID AT5G66900), *NRG1.2* (gene ID AT5G66910), and *NRG1.3* (gene ID AT5G66890). We further specified that MINORg should design these gRNAs such that they are usable in all 64 accessions included in a publicly available *A. thaliana* panNLRome resource (26). We retrieved the panNLRome data from http://ftp.tuebingen.mpg.de/ebio/alkeller/pan_NLRome/. As *NRG1.3* is known to antagonise neighbouring *NRG1.1* and *NRG1.2* (27), we decided to design gRNA for *NRG1.3* separately from the other two genes. The following command was used to run MINORg to design gRNA for *NRG1.1* and *NRG1.2*:


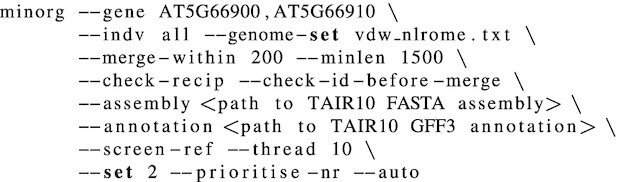


Using ‘- -gene’, we specified the gene IDs of *NRG1.1* and *NRG1.2* as our target genes. ‘- -genome-set’ tells MINORg the location of a lookup file that maps aliases to query FASTA files, which in this case are the contig-level assemblies of the panNLRome of 64 *A. thaliana* accessions, and ‘- -indv all’ indicates that all FASTA files listed in the lookup file are to be queried. A template of ‘vdw_nlrome.txt’ can be found at https://github.com/rlrq/MINORg/publication_data. ‘- -merge-within’, ‘- -minlen’, ‘- -check-recip’, and ‘- -check-id-before-merge’ are parameters that control homologue discovery. ‘- -thread’ informs the maximum number of parallel processes. All other parameters (gRNA length, PAM, restricting gRNA to CDS regions, 30% ≤ GC ≤ 70%, and 0 mismatch off-target threshold) were left as default.

MINORg identified 129 homologues in 64 accessions. The first set of gRNA output by MINORg comprised of only one gRNA (shared by all homologues), while the second comprised of two (one of which covered 113 homologues and the other covering the remaining 16). For the knockout experiment in Col-0, we selected the gRNA that could target both of Col-0’s homologues from the second set for cloning.

For *NRG1.3*, we repeated the above code with the exception of two parameters, ‘- -gene’ and ‘- -minlen’, which were replaced with ‘- -gene AT5G66890’ and ‘- -minlen 50’ respectively. MINORg identified 63 homologues in 64 accessions. As the positions of the gRNA from the first two sets overlapped, we selected the first set (comprising of one gRNA) and replaced the second set (also comprising of one gRNA) with a non-overlapping gRNA chosen from those with equivalent coverage as listed in the ‘_pass.eqv’ file. See Supplementary Table S2 for gRNA sequences.

### Molecular cloning for *RPW8/HR4* knockout

The subcloning of gRNAs into CRISPR-Cas9 vector pKI-1.1R (28) are detailed in our subcloning protocol (Supplementary Methods). To eliminate the possibility of the off-targeting of one gRNA editing the target of another gRNA, each plant individual was transformed with a CRISPR-Cas9 vector containing only one gRNA.

### A PCR-free modular cloning system for multiplexing CRISPR-Cas9 gRNA

**Figure 2.**
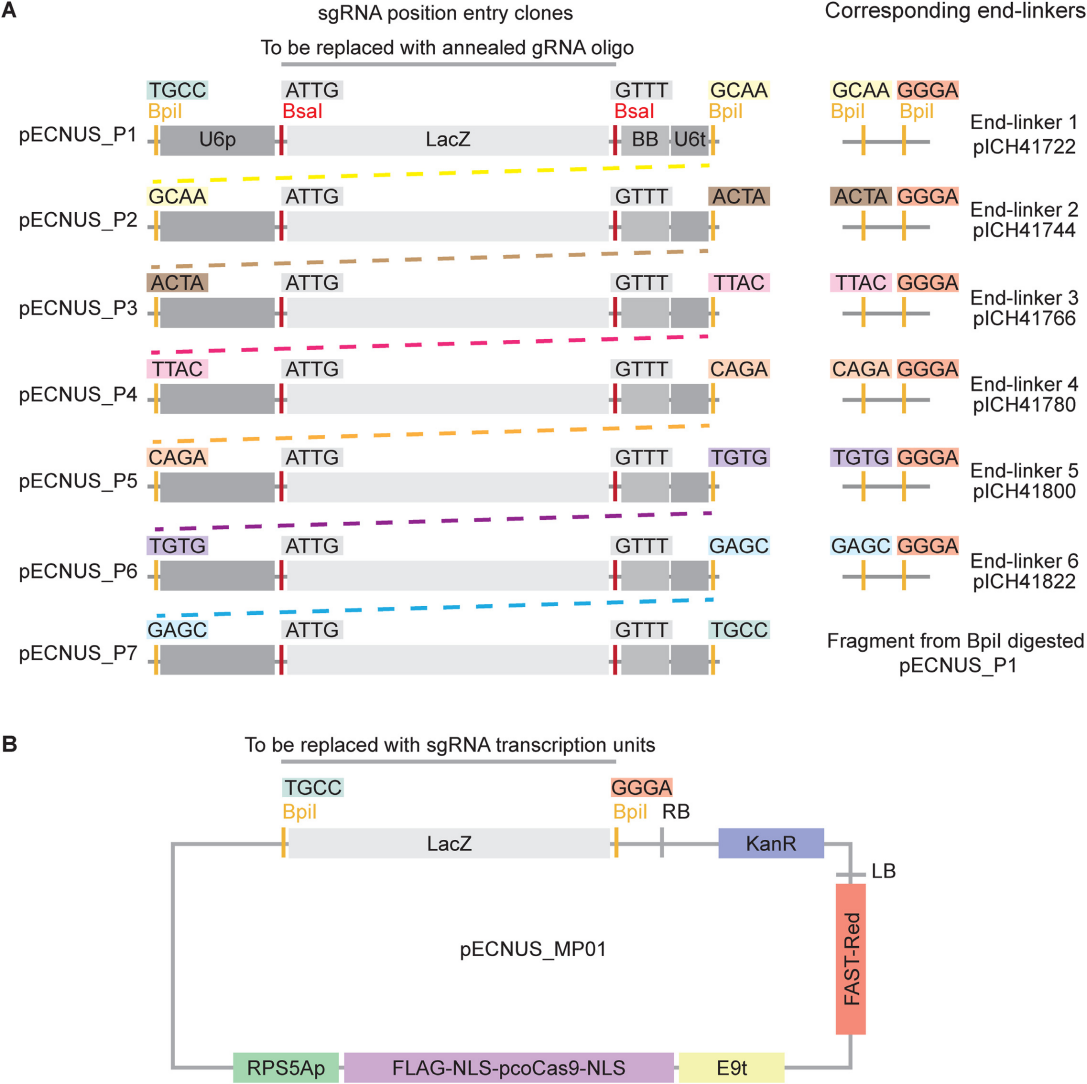
Overview of the modular cloning system for multiplexing CRISPR-Cas9 gRNA. (**A**) Schematic representation of sgRNA position entry position clones (pECNUS_P1 to pECNUS_P7) and their corresponding end-linkers. The sgRNA position clones incorporate an sgRNA expression cassette with a different overhang each and serve as intermediate shuffle-in vectors for final multiplexing vector assembly. The overhangs to be generated by BsaI or BpiI digestion are labelled above the respective enzyme recognition sites. With the exception of the restriction site overhangs, all other features, which are shared by all position entry clones, are labelled only in pECNUS_P1. BsaI digestion of all position entry vectors for the excision of LacZ and insertion of gRNA generates the same overhangs. Note that pECNUS_P7’s second BpiI overhang is complementary with pECNUS_P1’s first BpiI overhang. All end-linkers were previously described by Weber *et al.* (29). U6p: U6 promoter; BB: sgRNA backbone; U6t: U6 terminator. (**B**) Schematic representation of the final CRISPR multiplexing binary acceptor vector pECNUS_MP01. Overhangs generated by BpiI digestion are complementary with pECNUS_P1 (TGCC) and all six end-linkers (GGGA). RPS5Ap: RPS5A promoter; pcoCas9: plant codon optimised Cas9; E9t: E9 terminator; FAST-Red: transgenic plant selection marker; KanR: kanamycin resistance for bacterial selection; LB: T-DNA left border; RB: T-DNA right border. All vectors in the CRISPR-Cas9 multiplexing toolkit have been deposited at Addgene (Supplementary Table S3). Addgene numbers: 194343 (pECNUS_P1), 194344 (pECNUS_P2), 194345 (pECNUS_P3), 194346 (pECNUS_P4), 194347 (pECNUS_P5), 194348 (pECNUS_P6), 194349 (pECNUS_P7), 194350 (pECNUS_MP01), 194351 (pECNUS_MP02; not shown in this figure).

To facilitate multiplexed gRNA cloning, we designed a two-step modular cloning system by adapting a modular cloning system that utilises Golden Gate assembly (29,30) (Figure 2). By pre-assembling reusable components into entry vectors, we simplified the cloning process by reducing the number of separate units that need to be simultaneously combined through ligation. The system consists of two types of vectors (Supplementary Table S3): sgRNA position entry vectors (pECNUS_P1 to pECNUS_P7) (Figure 2 A) and binary vector (pECNUS_MP01 (Figure 2 B) and pECNUS_MP02 (Supplementary Figure S1)). We generated the seven position entry vectors by pre-assembling an sgRNA expression cassette into each of them. The cassette includes Arabidopsis U6 promoter, sgRNA backbone, and Arabidopsis U6 terminator. All position clones generate the same overhangs when digested by BsaI (ATTG and GTTT) for replacement of LacZ with gRNA, but different overhangs when digested by BpiI. pECNUS_MP01 and pECNUS_MP02 are binary vectors, derived from pAGM4723 (30), that we pre-equipped with an optimal Cas9 expression cassette: RPS5A promoter (RPS5Ap) (28), plant codon optimized Cas9 (pcoCas9) (31), and E9 terminator (E9t). Also included in the vector are LacZ flanked by BpiI restriction sites, and plant selection marker FAST-Red (32). The binary vectors differ only in the recognition site for overhang generation (BsaI for pECNUS_MP01 and BpiI for pECNUS_MP02).

The combination of elements in the Cas9 expression cassette in the position entry clones exhibited the best editing performance in *A. thaliana* in a screen conducted by (33). The FAST-Red selection marker renders the coat of successful transformed seeds a fluorescent red, thus enabling the positive and negative selection of CRISPR transgenes and simplifying the selection procedure of transgene-free mutants in the T_2_ or subsequent generations. In addition, the Cas9 and sgRNA expression cassettes are designed to be assembled in a head-to-head configuration in the final binary vector, as this arrangement had been shown to improve editing efficiency (33). Using this system, up to six sgRNA cassettes can be assembled into a final binary vector within two rounds of Golden Gate assembly.

The multiplexing system comprises of three major steps, none of which requires PCR. First, single-stranded forward and reverse oligonucleotides that encode a gRNA with appropriate overhang sequences are annealed to form double-stranded gRNA with overhangs for Golden Gate assembly. Next, position entry clones are digested by BsaI to excise LacZ and generate overhangs, and the linearised vectors are incubated with the double-stranded annealed gRNA with appropriate overhang sequences in order to generate sgRNA expression cassettes. Digestion and ligation can be simultaneously carried out with a one-tube one-step reaction. Finally, sgRNA expression cassettes that are to be combined into a single binary vector are excised by BpiI digestion from their respective sgRNA position entry clones to generate sgRNA expression cassettes with different overhangs. These cassettes and corresponding MoClo end-linkers (Figure 2) (30) are then tandemly ligated and inserted into BpiI-digested final binary vector pECNUS_MP01 to form the final CRISPR binary vector. This final digestion and ligation step can also be performed simultaneously in a single tube. As BsaI and BpiI are used to assemble the constructs, gRNA should not contain BsaI or BpiI recognition sites.

As described earlier, this multiplexing CRISPR cloning system is capable of assembling up to six sgRNA casettes into a final binary vector within two cloning steps. However, it can also be combined with the multi-gene MoClo system to introduce an additional layer of multiplexing using level M vectors (30,34) with pECNUS_MP02 as a final acceptor vector (Supplementary Table S3, Supplementary Figure S1) to clone up to 36 gRNA with only one additional round of Golden Gate assembly. This is achieved by assembling up to six gRNA cassettes in a single level M vector, following which multiple level M vectors can be assembled into the final binary vector pECNUS_MP02. As pECNUS_P7’s second overhang is complementary to pECNUS_P1’s first overhang (Figure 2), pECNUS_P7 can be tandemly assembled with pECNUS_P1 in level M vectors. Several examples of vector combinations that can be used to assemble different numbers of gRNA cassettes are detailed in Supplementary Table S4, and a visual schematic of how to assemble eight gRNA cassettes is provided in Supplementary Figure S2. Thus, this system of vectors that are pre-equipped with static, reusable elements is a suitable cloning strategy for efficient assembly of highly multiplexed cassettes of gRNA sets generated by MINORg.

### Multiplex cloning for *NRG1.1/2/3* knockout

All four gRNA designed for *NRG1.1/2/3* knockout were cloned using the multiplex CRISPR cloning strategy outlined above (Figure 2). gRNA oligos were designed with 5’ ATTG overhangs on the forward strand and 5’ AAAC overhangs on the reverse strand (Supplementary Figure S2). Annealing of gRNA oligos was conducted with 1 µM of forward oligos, 1 µM of reverse oligos, 1 µl of T4 ligase buffer (NEB #M0202S), topped up with distilled water to 10 µl. The mixture was incubated at 37 °C for 30 min, then 95 °C for 5 min, allowed to cool down at a rate of 5 °C/min to 25 °C and held at 25 °C for 5 min, and finally kept at 4 °C until use.

gRNA_110, gRNA_132, gRNA_045 and gRNA_041 were then inserted into position entry clones pECNUS_P1, pECNUS_P2, pECNUS_P3 and pECNUS_P4 respectively by simultaneous digestion and ligation (Supplementary Figure S2). This was carried out using 0.1 mg/ml of bovine serum albumin (BSA) (Biobasic #D0024), 1.5 µl of 10× T4 ligase buffer (Biobasic, #9K-005-0002), 10 U of BsaI-HF-v2 (NEB #R3733L), 200 U of T4 DNA ligase (Biobasic #9K-005-0002), 0.02 pmol of position clone entry vector, 0.0033 µM of annealed oligos, topped up with distilled water to 15 µl. The mixture was cycled through 20 cycles of 37°C for 5 min and 16°C for 5 min, followed by a single cycle of 50°C for 5 min and 80°C for 5 min, and finally held indefinitely at 16°C. The product was transformed into DH5α by heat shock at 42°C for 45 s and plated onto LB plates supplemented with kanamycin at 50 µg/ml. Blue-white selection was used to screen for clones with successful insertion. We verified correct insertion by Sanger sequencing.

Using the fourth end-linker (derived from ICH41780) to bridge overhang of the fourth position clone with that of the binary vector, all four position clones were tandemly assembled into pECNUS_MP01 to generate the final binary vector by simultaneous digestion and ligation again (Supplementary Figure S2). The reaction mixture comprised of 0.1 mg/ml of BSA (Biobasic #D0024), 1.5 µl of 10x T4 ligase buffer (Biobasic, #9K-005-0002), 5 U of BpiI (Thermo Fisher #ER1011), 200 U of T4 DNA ligase (Biobasic #9K-005-0002), 0.02 pmol of each of the four position clones with gRNA inserted, 0.06 pmol of vector pICH41800 containing the fourth end-linker, 0.02 pmol of pECNUS_MP01, topped up with distilled water to 15 µl. The mixture was cycled through 35 cycles of 37°C for 5 min and 16°C for 5 min, followed by a single cycle of 50°C for 5 min and 80°C for 5 min, and finally held indefinitely at 16°C. Correct insertion was once again verified by Sanger sequencing.

### Plant transformation

Binary vectors were transformed into *Agrobacterium tumefaciens* strain GV3101 and subsequently into the relevant *A. thaliana* accessions according to Hoffgen and Willmitzer (35). The T_1_ generation was sown on $\frac{1}{2}$ MS plates. MS plates for *RPW8/HR4* transformants were supplemented with hygromycin (15 μg/ml) for selection. T_1_*NRG1.1/2/3* transformants were selected by fluorescence microscopy using RFP filter before sowing.

### DNA extraction

For *RPW8/HR4* transformants, leaf tissues were harvested from resistant plants and genomic DNA was extracted with Edwards buffer made in-house (36). For *NRG1.1/2/3* transformants, leaf tissues were harvested from 3-week old plants and genomic DNA was extracted using the CTAB plant genomic DNA extraction method (37).

### Deep-sequencing of NGS reads

We assessed the editing status of each locus by deep-sequencing via Illumina iSeq 100. The procedure involves three rounds of PCR: (i) the first PCR generated an amplicon of size 526–2254 bp that includes the CRISPR-Cas9 cleavage site. For the *RPW8/HR4* knockout experiment, primers for the first PCR aims to amplify as few *RPW8/HR4* members as possible (ideally, one but it is not always possible if the homologues are identical, especially at Primer3-optimal sites). (ii) Next, the second PCR amplified a 250–280 bp region covering the cleavage site for each *RPW8/HR4* or *NRG1* member. The second PCR primers consist of 5′ adapter sequences to which the (iii) primers of the third PCR binds to append iSeq index sequences. All gRNA and primer sequences are deposited in Supplementary Tables S1 (*RPW8/HR4*) and S2 (*NRG1.1/2/3*).

### Amplicon primer design for *RPW8/HR4* knockout

In TueWa1-2, members of *RPW8.1* and *RPW8.2* are duplicated and the remaining *RPW8* members share high sequence similarity even in intergenic regions. With a large number of *RPW8* members (10 genes in TueWa1-2), the manual design of theoretically optimized primers that specifically amplify each gene is challenging. In addition, specific primers were not always available, thus at certain regions, the first or second PCR amplicons generated may consist of sequences of two or more *RPW8* members. In such cases, the next acceptable solution was to use polymorphic sites to differentiate the amplicons/NGS reads per gene. For every MINORg-mediated CRISPR-Cas experiment, we foresee this complex process of primer design on a continuous genome is repeated for each new gene cluster targeted, which indicates that this tedious work can be automated to significantly save time.

To solve the primer design issue, we wrote and used a programme called ‘PRIMERg’ (https://github.com/CherWeiYuan/primerg). PRIMERg takes a list of gRNA and a genomic template sequence and returns primers for the first and second PCR. Primers provided by PRIMERg are optimized by primer3 and filtered, if possible, by the specificity within the user-supplied genomic template. The specificity of these primers was checked by a homebrewed algorithm based on the Primer-BLAST algorithm (17). The uniqueness (whether there are distinctive SNP(s) present in the desired amplicon) for each primer is checked by string matching against the user-supplied genomic template.

For certain genes, specific first PCR primers cannot be designed, hence we rely on the uniqueness of each amplicon to differentiate the reads from different genes. Such unique SNPs can be detected by aligning the desired and undesired amplicons. For our case, in TueWa1-2, the region flanking *RPW8.1a* + *RPW8.3b* and *RPW8.1a*_1 + *RPW8.3c* is highly similar and all suitable primer3-optimized primer pairs amplified the two regions, each consisting of two genes. To obtain the reads for *RPW8.3b*, we wrote a Python function to select reads with the signature of *RPW8.3b* (‘cttaagacgttcac’, not present in *RPW8.1a/8.1a_1* or *RPW8.3c*), with an allowance of 1-bp mismatch to account for sequencing error (https://github.com/CherWeiYuan/SNP_Filtering). We then mapped the filtered reads to the amplicon and visualized the results using IGV (38) to check for any discrepancies (e.g. unexpected SNPs that suggest undesired amplicons are also mapped). The clean reads were used for editing rate analysis.

To increase the number of samples we include per run in our iSeq 100 (Illumina), we allowed amplicons from different genes to share the same sample indices. The desired amplicon was also selected from the pool of amplicons with the same index via the presence of unique SNPs before IGV mapping and CRISPResso2 analysis as described above. More specifically, to select TueWa1-2 *RPW8.3a* reads without KZ10 *RPW8.3* reads, we filtered R1 reads by ‘aatagaaatacat’ and R2 reads by ‘acaatcgat’. To select TueWa1-2 *RPW8.2b* reads without KZ10 *RPW8.3* reads, we filtered the R1 reads by ‘gttctcaagg’.

### Analysis of indels by CRISPResso2

Reads were fed to CRISPResso2 (39) (settings: ‘Minimum average read quality (phred33 scale)’ > 30, ‘Minimum single bp quality (phred33 scale)’ > 10). Gap characters were counted for columns Aligned_Sequence and Reference_Sequence in the output file Alleles_frequency_table_around_sgRNA_*.txt, and gaps in Aligned_Sequence were subtracted from those in Reference_Sequence for each allele alignment to obtain net insertions around the gRNA editing site for each allele. Alleles with non-zero net insertions were considered edited.

### Analysis of structural rearrangement for *NRG1.1/2/3* knockout

As *NRG1.1/2/3* are tandemly located within 7.8 kb, we sought to detect chromosome rearrangement events induced by the four gRNA. Rearrangements were analysed by PCR genotyping using primers flanking the chromosome regions of interest with wildtype sample as negative control. See Supplementary Table S2 for primers used. For validation, we extracted five PCR product bands using Bio Basic’s PCR purification kit and sent them for Sanger sequencing.

### Design of pangenomic Cas12a gRNA for the NB-ARC domain of *TN3* using MINORg

To demonstrate MINORg’s ability to design gRNA for a specific domain of orthologous genes in a pangenome, we designed gRNA to target the NB-ARC domain (40) of *TIR-NBS3* (*TN3*; gene ID AT1G66090). Additionally, we show MINORg’s versatility with respect to Cas systems by designing gRNA for Cas12a (Cpf1), which has a 5’ TTTV PAM and a standard gRNA length of 23 nucleotides (41) as compared to Cas9’s 3’ NGG PAM and standard 20 nucleotides gRNA (42,43).

To design gRNA for *TN3* orthologues in the *A. thaliana* panNLRome, we ran the following code:


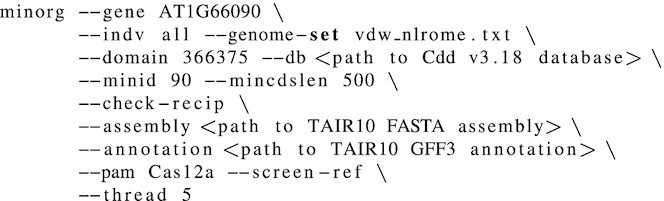


Using ‘- -gene’, we specified AT1G66090 (*TN3*’s gene ID) as our target gene. As with the *NRG1* example above, ‘- -genome-set’ and ‘- -indv all’ tell MINORg to search for homologues in a panNLRome resource of 64 *A. thaliana* accessions (26). ‘- -db’ specifies the path to a local CDD database (version 3.18; previously retrieved from ftp://ftp.ncbi.nih.gov/pub/mmdb/cdd/cdd.tar.gz but has since been superseded), and ‘- -domain’ specifies the position-specific scoring matrix (PSSM) ID of the domain to be targeted, which in this example is the NB-ARC domain. ‘- -minid’, ‘- -mincdslen’, and ‘- -check-recip’ are parameters that control homologue discovery. With ‘- -pam’, we specified the 5’ TTTV PAM of Cas12a (41), asked MINORg to generate 23 bp gRNA using ‘- -length 23’, and ‘- -thread’ informs the maximum number of parallel processes. All other parameters (restricting gRNA to CDS regions, 30% ≤ GC ≤ 70%, and 0 mismatch off-target threshold) were left as default.

After removing all entries for the potentially non-functional MNF-Che-2 homologue of *TN3* from the mapping file (ending in ‘_gRNA_all.map’) output by MINORg, we used the following code to regenerate gRNA sets for the reduced list of targets:


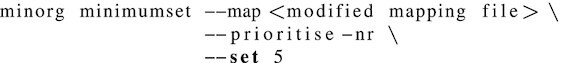


‘- -prioritise-nr’ tells MINORg to prioritise non-redundancy over proximity to 5’ end when generating gRNA sets.

### Phylogenetic inference of NB-ARC domains of *TN3* orthologues

In the course of executing MINORg for the generation of pangenomic gRNA sets for *TN3*, an alignment of non-reference targets with reference genes was generated by MAFFT (18). We fed this alignment to FastTree (44) using ‘- -nt’ for nucleotide sequence and default parameters otherwise to generate a maximum-likelihood tree.

### Design of inter-species, multi-PAM gRNA for *ADR1* and *NRG1.1* using MINORg

To demonstrate how MINORg can be used to design cross-species gRNA, we designed gRNA for conserved immune genes, *ACTIVATED DISEASE RESISTANCE 1* (*ADR1*) and *NRG1.1*, in three Arabidopsis species: *A. thaliana*, *Arabidopsis lyrata* and *Arabidopsis halleri*. Furthermore, we also show how to specify a non-standard, non-preset PAM pattern, as well as multiple PAMs.

We retrieved reference genome assemblies and GFF3 annotations for *A. thaliana* (TAIR10), *A. lyrata* (version 2.1; GenBank assembly accession GCA_000004255.1; retrieved from ftp://ftp.ensemblgenomes.org/pub/plants/release-45/fasta/arabidopsis_lyrata), and *A. halleri* (version 1.1; retrieved from https://data.jgi.doe.gov/refine-download/phytozome?organism=Ahalleri&expanded=264), and ran the following code:


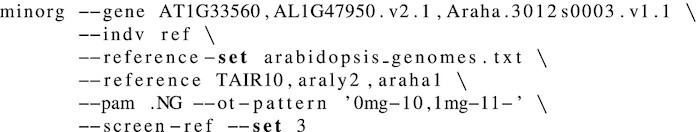


Using ‘- -gene’, we specified the gene IDs of our target genes (AT1G33560 is the gene ID for *ADR1* in *A. thaliana*, AL1G47950.v2.1 in *A. lyrata*, and Araha.3012s0003.v1.1 in *A. halleri*). ‘- -reference-set’ tells MINORg the location of a lookup file that maps reference genome aliases to assembly and annotation combinations, while ‘- -reference’ specifies the aliases of reference genomes to use. ‘- -pam .NG’ specifies a non-standard 3’ NG PAM pattern. ‘- -ot-pattern ’0mg-10,1mg-11-’’ instructs MINORg to exclude gRNA with at least one off-target hit that has no mismatches or gaps between positions –1 and –10 (0mg-10) and no more than one gap or mismatch from position –11 to the 5’ end of the gRNA (1mg-11-). All other parameters (including 20 bp gRNA length, restricting gRNA to CDS regions, and 30% ≤ GC ≤ 70%) were left as default.

The above code was repeated using ‘- -gene AT5G66900,AL8G44500.v2.1,Araha.11408s0003.v1.1’ to generate gRNA targeting *NRG1.1* orthologues, where AT5G66900, AL8G44500.v2.1, and Araha.11408s0003.v1.1 are gene IDs for *NRG1.1* in *A. thaliana*, *A. lyrata* and *A. halleri* respectively.

This section was repeated with ‘- -pam ’.(NG|NNG|GAA|GAT)’’, which specifies multiple PAM patterns (3’ NG, NGG, GAA, and GAT) that are recognisable by variants of xCas9 (evolved Cas9 variants with broadened PAM compatibility) (45) to demonstrate MINORg’s flexibility and compatibility with diverse Cas systems.

## RESULTS

### Multi-target edits in T_1_ generation of two *A. thaliana* accessions using three gRNA in single-plexed vectors

To validate the utility of gRNA output by MINORg, we attempted to knock out 13 homologous genes in two *A. thaliana* accessions (TueWa1-2 and KZ10) using gRNA generated by MINORg. *RESISTANCE TO POWDERY MILDEW 8* (*RPW8*) and *HOMOLOG OF RPW8* (*HR*) are immune genes in *A. thaliana* that comprise a physical cluster conferring broad-spectrum resistance to powdery mildew (46). The composition and number of *RPW8/HR* cluster members vary wildly between different *A. thaliana* accessions (25) due to a history of duplication and diversifying selection (47). In fact, the reference genome of the *A. thaliana* accession Col-0 lacks *RPW8* genes entirely. These features make the *RPW8/HR4* cluster ideal for testing MINORg-generated gRNA for multiple homologous genes in multiple individuals.

Using MINORg, we designed two mutually exclusive gRNA sets that are separately able to cover a subset of the *RPW8/HR* cluster consisting of all *RPW8* genes as well as *HR4* (henceforth collectively referred to as *RPW8/HR4*) in accessions TueWa1-2 and KZ10. TueWa1-2 has ten *RPW8/HR4* genes while KZ10 has three *RPW8* genes and no *HR4*. Both accessions also possess paralogous *HR1/2/3* genes within their *RPW8/HR* clusters, which serve as potential off-target risk. As neither accession has had its full genome sequenced, we performed an off-target assessment in the reference Col-0 genome, taking care to mask *HR4*, which is the only target gene also present in Col-0.

We subcloned six gRNAs (set1: gRNA_1022, gRNA_1023, and gRNA_1027 and set 2: gRNA_1033, gRNA_1034 and gRNA_1035) individually into CRISPR-Cas9 vectors, which were in turn transformed in individual plants. TueWa1-2 is known to have low transformation efficiency (48) and we obtained very few (n < 3) or no T_1_ plant transformants for gRNA_1022, gRNA_1027 and gRNA_1035; the few positive plant transformants did not have their genomes edited. The remaining gRNAs, although from different MINORg sets, was still able to target all TueWa1-2 and KZ10 *RPW8/HR4* genes. Specifically, gRNA_1033, which targets *RPW8.2/8.3* homologues, targeted six genes in TueWa1-2 (*RPW8.3a/3c’/2a/3b/2b/3c*) and two genes in KZ10 (*RPW8.2/8.3*). gRNA_1023 targets *RPW8.1* homologues, which were three genes (*RPW8.1a/1a_1/1b*) in TueWa1-2 and *RPW8.1* in KZ10. Lastly, gRNA_1034 specifically edited *HR4* in TueWa1-2, a gene that is missing in KZ10. The analysis for editing efficiency at 11/13 loci was completed (for the remaining two loci, *RPW8.2b* and *RPW8.3c* in TueWa1-2, deep-sequencing failed as primers designed for them amplified their homologues instead).

**Figure 3.**
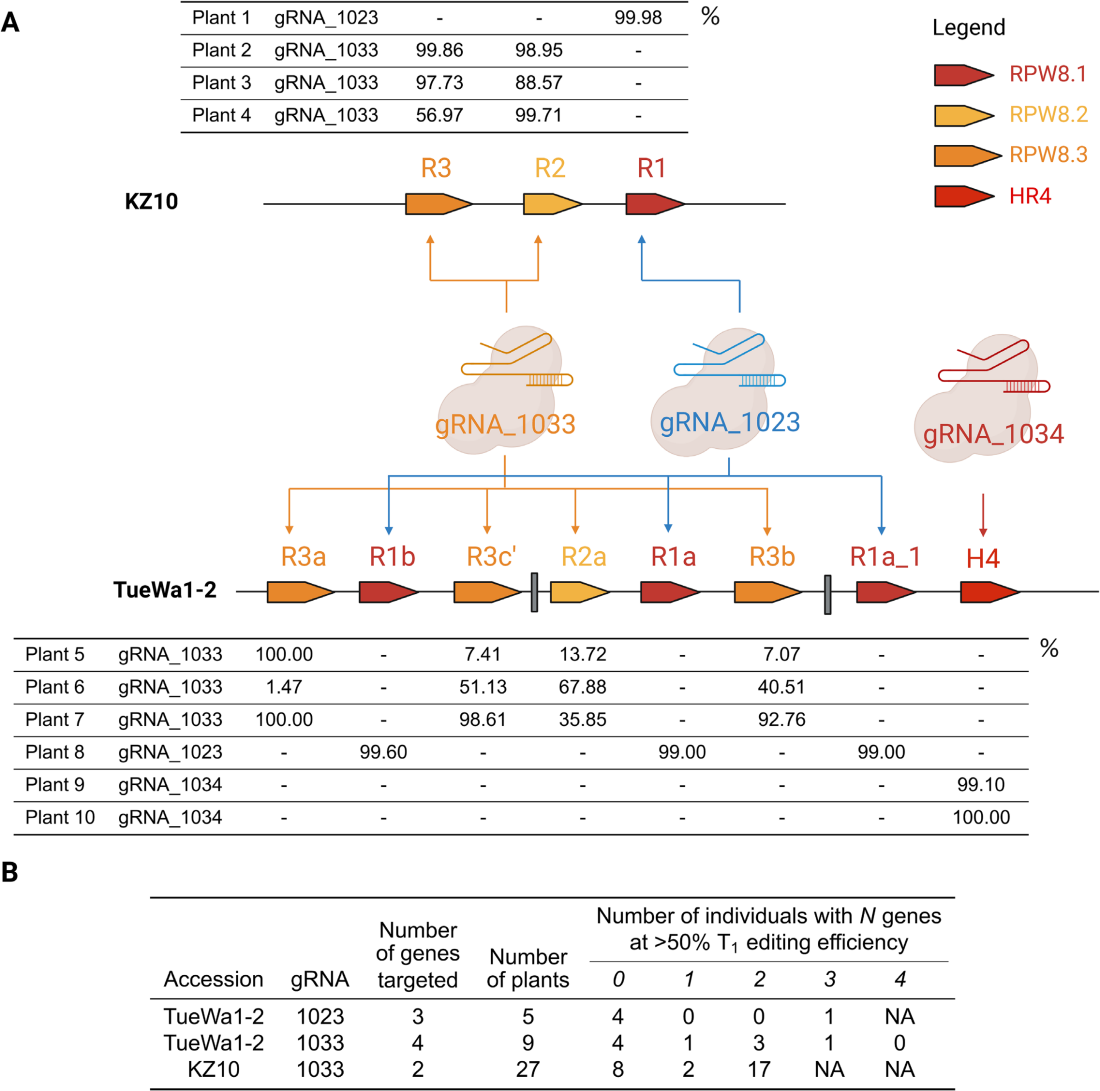
Editing efficiency of multi-gene targeting of the *RPW8/HR4* cluster in T_1_ plants. (**A**) Summary of gRNAs and their RPW8/HR4 targets in TueWa1-2 and KZ10. Every plant individual was transformed with a CRISPR-Cas9 vector containing one gRNA. The table show the percent of NGS reads with non-zero net inserted bases. Not shown are *RPW8.2b* and *RPW8.3c*, two of ten TueWa1-2 targets, for which deep sequencing failed. (**B**) Editing efficiency in T_1_ plants. Created with BioRender.com (excluding tables).

Overall, deep-sequencing revealed that 10 out of 11 genes were edited beyond 90% and the gene most resistant to editing (*RPW8.3b*) had an individual with 68% of the reads edited (Figure 3A, Supplementary Table S5). For individuals transformed with a gRNA targeting multiple genes (i.e. gRNA_1033 and gRNA_1023), we observed multiple genes edited within the same individual (Figure 3B, Supplementary Table S5). Most impressively, for TueWa1-2 plant 8 with gRNA_1023, all three *RPW8.1* homologues were edited beyond 99% (Figure 3B, Supplementary Table S5). For gRNA_1033, we observed TueWa1-2 plant 7 which had > 92% editing efficiency at three genes (*RPW8.3a/3c’/3b*); *RPW8.2a* was unfortunately edited at 7.54% but was edited at 68% in another individual, plant 6. For KZ10, editing efficiency was generally high (Figure 3B, Supplementary Table S5). Most editing events that resulted in indels were also frameshift mutations (Supplementary Table S5). We obtained only one transgenic plant for KZ10 with gRNA_1023, but the editing of *RPW8.1* was successful (99.9%). Three plants were obtained for KZ10 with gRNA_1033, which targeted two genes, and the mean editing efficiency was 90%.

### Multi-target edits in T_1_ generation of *A. thaliana* Col-0 using a multiplexed vector

**Figure 4.**
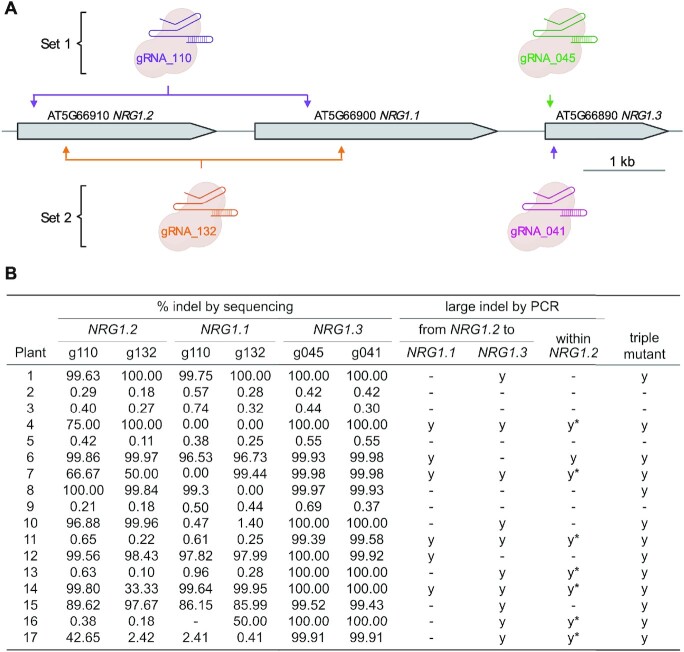
Editing efficiency of multi-gene targeting of *NRG1.1/2/3* in T_1_ plants using a multiplexed CRISPR construct. (**A**) Summary of two mutually exclusive sets of gRNA (set 1: gRNA_110 and gRNA_045; set 2: gRNA_132 and gRNA_041) that each target all *NRG1* genes (*NRG1.1/2/3*) and their editing sites. (**B**) Editing efficiency in Col-0 T_1_ plants, as detected by either deep-sequencing or PCR. For indels detected by PCR, those detected by positive and negative PCR results are indicated with ‘y’ and ‘y*’ respectively. An individual is classified as a triple mutant (‘y’) if every gene is either spanned by a PCR-detected indel or has at least one editing site with >90% indels as detected by sequencing. gRNA names are abbreviated by collapsing ‘gRNA_’ to ‘g’ (e.g. ‘gRNA_110’ is abbreviated as ‘g110’). Created with BioRender.com (excluding table).

To demonstrate multi-gene targeting using a multiplexed construct, we designed two sets of mutually exclusive gRNA for a tandem array of three *A. thaliana* genes: *NRG1.1*, *NRG1.2* and *NRG1.3* (Figure 4A). We cloned both sets into a single binary vector (Supplementary Figure S2) using our multiplexing CRISPR cloning system (Figure 2) and transformed the vector into Col-0. Editing rate was assessed in T_1_ plants by deep-sequencing and PCR (Figure 4B).

Extensive indels were observed in 13 out of 17 T_1_ genotyped individuals for *RPW8/HR4* knockout, and all editing sites of both sets of gRNA showed evidence of editing (Figure 4B, Supplementary Table S5). Similar to the *RPW8/HR4* cluster knockout, most editing events that resulted in indels were also frameshift mutations (Supplementary Table S6). In general, editing rates as detected by sequencing was uniform across all editing sites within an individual, except where an editing site is spanned by a large, PCR-detected indel, which likely interfered with the PCR of amplicons for deep-sequencing at the editing site (Figure 4). Using genotyping by PCR, we observed large fragment deletion beginning and ending at different combinations of gRNA editing sites in 13 individuals (Figure 4B). By Sanger sequencing of PCR products from five individuals, we confirmed the deletions and noted that all gRNA editing sites contributed to at least one large fragment deletion event (Supplementary Figure S4). Thus, we showed that our multiplexing CRISPR-Cas9 cloning system can achieve high editing efficiency, and that it can be used with gRNA output by MINORg for effective multi-gene knockout.

### Pangenomic gRNA design for orthologues in 64 *A. thaliana* accessions for a non-Cas9 system

**Figure 5.**
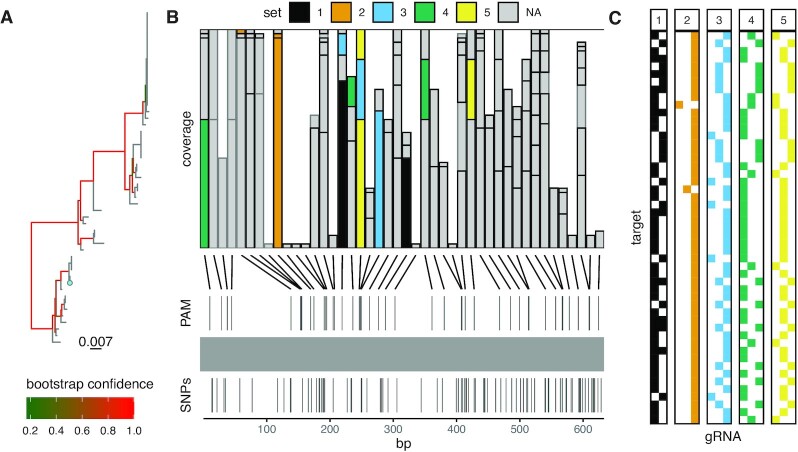
MINORg generates small sets of gRNA for pangenomic coverage of the NB-ARC domain of *TN3* in 51 *A. thaliana* accessions. (**A**) Maximum-likelihood tree of the genomic sequence of the NB-ARC domain of *TN3* orthologues in 51 *A. thaliana* accessions. The NB-ARC domain is contained within a single exon in all accessions. The reference accession, Col-0, is indicated in cyan. (**B**) Coverage of all possible Cas12a gRNA (5’ TTTV PAM) for the NB-ARC domain of 51 *TN3* orthologues in 51 accessions. gRNAs that share the same PAM site are stacked. The height of each bar represents the number of targets covered by a gRNA. The horizontal line marks the maximum coverage of targets per PAM site, which is 51 targets. gRNAs that passed all checks (GC content, off-target, and within CDS) are outlined in black, and those that failed at least one check are outlined in grey. Five mutually exclusive sets were requested, with priority given to non-redundancy, and the final selection of gRNAs is coloured by set. Each set is capable of covering all 51 targets. (**C**) Coverage of each gRNA in each of the five output sets. Each of the 51 rows represents one orthologue, and each column represents one gRNA. Coloured cells indicate that an orthologue is covered by the gRNA in the corresponding column. Set colours are shared with (B).

To demonstrate pangenomic gRNA design, we designed gRNA for *TN3*, an immune gene, in 64 *A. thaliana* accessions using a publicly available panNLRome resource (26). This resource was generated using resistance gene enrichment sequencing (RenSeq) of 64 diverse *A. thaliana* accessions and is to date the most comprehensive inventory of NLRs for *A. thaliana*. Using MINORg, we queried the panNLRome dataset and identified 52 orthologues of *TN3* in 51 of the 64 accessions, one accession of which (accession MNF-Che-2) had two homologues. We asked MINORg to design up to five sets of gRNA for Cas12a (Cpf-1) (49) systems to target the moderately conserved catalytic NB-ARC domain (Figure 5A), making sure we included the full panNLRome dataset as well as the reference genome for off-target assessment.

Upon manual inspection of the inferred targets, we noticed that one of MNF-Che-2’s homologues had six different frameshift indels, suggesting that it is non-functional. We removed this homologue from the mapping file that MINORg output. As it is inconsequential whether this non-functional homologue is cleaved by a gRNA targeting functional *TN3* homologues, we did not execute the ‘filter’ subcommand to reassess off-target effects with this homologue as background for the updated list of targets. Using the modified mapping file, we executed the ‘minimumset’ subcommand to regenerate gRNA sets based on this smaller set of 51 targets, and asked MINORg to prioritise non-redundancy within sets over proximity to the 5’ end. The first set output by MINORg comprised only of two gRNA, while the rest had three gRNA (Figure 5B, Supplementary Table S7). All sets are capable of covering all 51 targets (Figure 5C). This exemplifies MINORg’s ability to identify minimal gRNA panels that are nevertheless suitable for species-wide screens in a large number of lineages.

### Cross-species gRNA design for orthologues in three Arabidopsis species for a multi-PAM system

**Figure 6.**
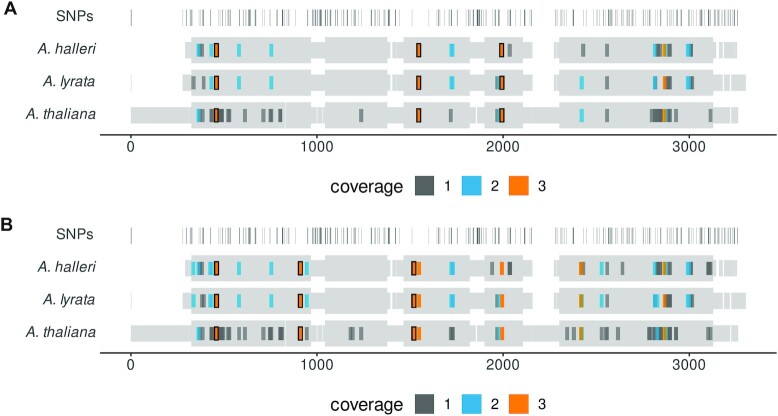
MINORg favours high coverage gRNA towards the 5’ end of *ADR1* and its orthologues in three Arabidopsis species. Multiple sequence alignment of genes Araha.3012s0003.v1.1 (*A. halleri*), AL1G47950.v2.1 (*A. lyrata*), and *ADR1* (*A. thaliana*) is shown in grey, with thicker sections representing coding regions. Single nucleotide polymorphisms (SNPs) are indicated in the first row. All candidate gRNA for 3’ NG PAM (**A**) and a multi-PAM xCas9 variant (which has broadened PAM compatibility and recognises NG, NNG, GAA, and GAT) (**B**) generated by MINORg within CDS regions that have passed off-target checks and contain GC content between 30% and 70% are shown along each gene. The colour of each gRNA corresponds with the number of orthologues it is capable of targeting. Three sets of gRNA were requested, and MINORg output three mutually exclusive sets that each contained only a single gRNA capable of covering all three orthologues for both NG PAM and xCas9 PAMs. These gRNA are outlined in black.

To demonstrate cross-species gRNA design, we designed gRNA for conserved immune genes *ADR1* and *NRG1.1* in three Arabidopsis species, *A. thaliana*, *A. lyrata* and *A. halleri*. We asked MINORg to design up to three mutually exclusive gRNA sets within coding regions for each gene and its orthologues using a non-standard 3’ NG PAM, as well as a variants of xCas9 (evolved Cas9 variants with broadened PAM compatibility) that recognise 3’ NG, NGG, GAA and GAT as PAM sequences (45). MINORg was also instructed to exclude any gRNA which has at least one off-target hit with no mismatches or gaps in the 10 bases proximal to the PAM site and up to 1 mismatch or gap from the 11th position onward. MINORg output three sets containing one gRNA each covering all three orthologues for both *ADR1* (Supplementary Tables S8 [NG PAM] and S9 [xCas9]) and *NRG1.1* (Supplementary Tables S10 [NG PAM] and S11 [xCas9]). Figure 6 shows candidate gRNA for *ADR1* orthologues Araha.3012s0003 (*A. halleri*), AL1G47950 (*A. lyrata*), and AT1G33560 (*A. thaliana*) that have passed all filtering checks, as well as the three gRNA output by MINORg for both NG PAM and multi-PAM xCas9 that are each capable of targeting all three orthologues. MINORg notably favours not only high coverage gRNA but also gRNA closer to the 5’ end in order to increase the likelihood that indels would have deleterious effects. By demonstrating MINORg’s ability to design inter-specific gRNA in addition to intra-specific gRNA (Figure 3), and further highlighting MINORg’s compatibility with atypical PAMs, we show that MINORg is highly flexible and can be used to design gRNA for diverse CRISPR experimental designs.

## DISCUSSION

In the pangenome era, the research community has access to a continually updated database of non-reference genomes. Currently, in *A. thaliana*, the contig-level assemblies of the panNLRome of 64 accessions (26) are publicly available. In response to the demand of pangenome tools, particularly in the functional investigation of gene or their clusters in non-reference genomes, we wrote MINORg, a powerful and versatile tool that facilitates inter-accession, multi-gene and minimal set gRNA design. We tested the minimal set targeting on 13 *RPW8/HR4* genes across two accessions and confirmed the successful editing in 11 of them with the expected multi-gene targeting within the same individuals observed.

In plants with a gRNA (i.e. gRNA_1033/ gRNA_1023) targeting multiple genes, we observed high T_1_ editing efficiency of single genes (Figures 3, 4, Supplementary Tables S5 and S6). Our data indicate that a single gRNA can be used to target as many as four genes of which we can expect three to be highly edited in T_1_ somatic cells. As the level of mosaicism in T_1_ plants is strongly correlated to the proportion of T_2_ and T_3_ homozygous progenies (50,51), it is likely that our genome edits are transgenerational. It is pertinent that the number of genes we can target is not limited by MINORg, but rather the wet lab genome editing tools used.

It is known that Cas9 is a limiting factor in plant multiplex applications (52). To overcome this, we designed a multiplexing CRISPR-Cas9 cloning system (Figure 2) to increase the probability of getting more genes highly edited within the same genome. We demonstrated the combined utility of MINORg and our cloning system by knocking out tandemly arranged genes *NRG1.1/2/3* using gRNA designed by MINORg that have been cloned into a single multiplexing vector pECNUS_MP01. All gRNA sites were highly edited in the T_1_ generation, and extensive indels spanning different combinations of gRNA editing sites were also observed, thus demonstrating editing efficiency. Depending on the proximity of targets to each other and whether such structural rearrangement is desired, we advise using MINORg with a carefully curated list of targets to avoid or enhance the likelihood of such editing events. Alternatively, a low-efficiency CRISPR-Cas system can be employed to temporally spread out editing events.

We have thus shown that MINORg can be used to generate sets of a small number of gRNA capable of targeting a larger number of homologous genes in multiple genetic backgrounds within the same species. Additionally, we also demonstrated that MINORg can be used to design gRNA for inter-species orthologues (Figure 6, Supplementary Tables S8–S11). In the absence of genome sequencing data for non-reference individuals of a species, users may take advantage of MINORg’s prioritisation of high coverage gRNA to design inter-species gRNA of orthologous genes in reference genomes of closely related species, as the conserved regions targeted by gRNA with high inter-species coverage are likely also conserved in those non-reference individuals. Furthermore, we showed that MINORg is capable of designing gRNA for non-Cas9 systems, such as Cas12a (Figure 5, Supplementary Table S7) and multi-PAM xCas9 (Figure 6, Supplementary Tables S9 and S11). All this further illustrates MINORg’s versatility to investigate genes not present in the reference genome with diverse CRISPR-Cas systems. While several other tools have a subset of some of MINORg’s features, none so far accommodate all of them (Supplementary Table S12).

**Figure 7.**
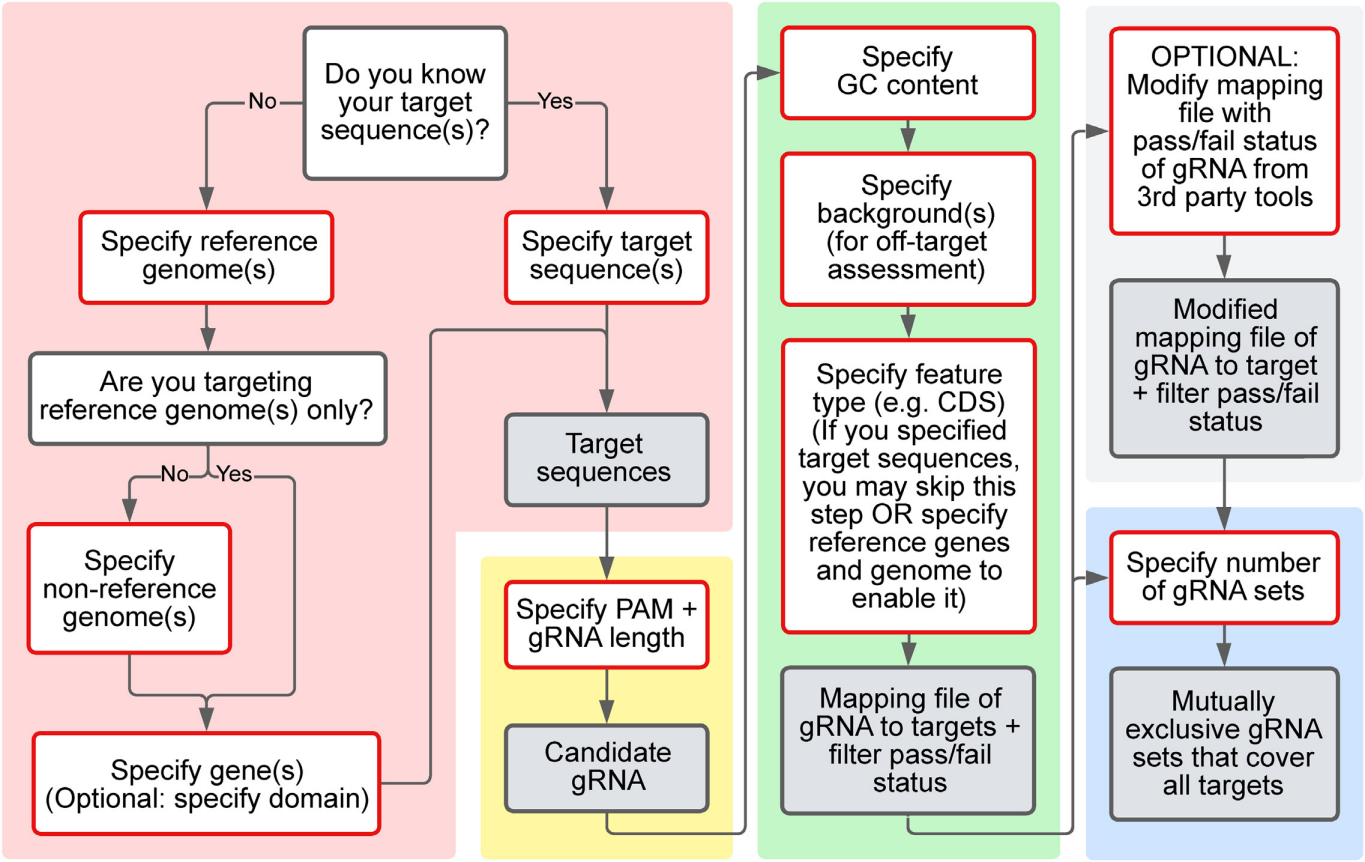
MINORg parameter selection flowchart. The flowchart is separated into four sections by background colour that correspond to each of the four main steps of MINORg described in Figure 1: target identification (pink), gRNA identification (yellow), gRNA filtering (green), and generation of minimum set (blue). Light grey background indicates optional integration of on- and off-target results from other tools. Boxes outlined in red describe parameters to use, and boxes with grey fill are the output of each step.

In sum, MINORg is a flexible gRNA design tool ideal for the pangenome era, as it accounts for both sequence variation as well as genetic background. It can be further supplemented with our multiplexing CRISPR-Cas9 cloning system to enhance simultaneous multi-gene editing. In Figure 7, we provide a flowchart of the basic functionalities of MINORg to give an idea of how MINORg can be customised to design gRNA for multiple targets with sequence homology in multiple genomes.

## DATA AVAILABILITY

Source code is freely available at: https://github.com/rlrq/MINORg (MINORg) and https://github.com/CherWeiYuan/primerg (PRIMERg). Documentation of MINORg, including tutorial and more detailed overview of sub-command algorithms, can be found at: https://rlrq.github.io/MINORg. Scripts and data are deposited at https://github.com/rlrq/MINORg/tree/master/publication_scripts and https://github.com/rlrq/MINORg/tree/master/publication_data. MINORg can be installed via Python’s package installer pip from the PyPI repository under the package name ‘minorg’. MINORg is also available as a Docker image as ‘rlrq/minorg’ (bundled with Cdd database version 3.18) and ‘rlrq/minorg-lite’ (without Cdd database). Fastq files for amplicon sequencing are deposited at the Dryad repository with the dataset identifier https://doi.org/10.5061/dryad.5dv41ns9r.

## Supplementary Material

gkad142_Supplemental_Files
