# Supplementary material for: Generating minimum set of gRNA to cover multiple targets in multiple genomes with MINORg
Source: Nucleic Acids Res. 2023 Mar 15;51(8):e43. doi: 10.1093/nar/gkad142 (PMC10164578; doi:10.1093/nar/gkad142)
Supplement: gkad142_Supplemental_Files [file gkad142_supplemental_files.zip › Sup_minorgManuscript_v4_NAR.pdf]

Generating minimum set of gRNA to cover multiple targets in multiple genomes with MINORg: Supplementary materials

Rachelle R.Q. Lee [1], Wei Yuan Cher [1], Jinge Wang [1], Yujie Chen [1,2], Eunyoung Chae [1*]

January 27, 2023

[1]Department of Biological Sciences, National University of Singapore, Singapore 117558
[2]School of Life Science and Technology, Xi'an Jiaotong University, Xi'an, Shaanxi 710049, P. R. China

[*]To whom correspondence should be addressed. Email: dbsce@nus.edu.sg

1 Supplementary Tables

Table S1. CRISPR multiplexing toolbox. Includes Addgene numbers of our vectors and names of vectors from which our vectors were derived.

Table S2. CRISPR multiplexing examples.

Table S3. gRNA and primers for *RPW8/HR4* knockout.

Table S4. gRNA and primers for *NRG1.1/2/3* knockout.

Table S5. Indel and frameshift rates for *RPW8/HR4* knockout. NGS reads were aligned to amplicons by CRISPResso2. Alignments of reads to reference sequence around editing sites were retrieved from the CRISPResso2 output file 'Alleles_frequency_table_around_sgRNA_*.txt'. n_reads: total number of reads aligned; n_indel: number of reads with gaps in either read or reference alignment; n_net_insertion: number of reads where gaps in read alignment (deletions) is not equal to gaps in reference alignment (insertions); n_net_insertion_frameshift: number of reads where n_net_insertion is not a multiple of 3; p_net_insertion_is_frameshift: percentage of n_net_insertion reads that are also n_net_insertion_frameshift reads (that is, frameshift events as a percentage of non-zero net insertion events). All other columns prefixed with 'p_' are percentages (relative to n_reads) of their corresponding columns prefixed with 'n_'.

Table S6. Indel and frameshift rates for *NRG1.1/2/3* knockout. See Table S5 for table description.

Table S7. gRNA and their coverage for pangenomic *TN3* gRNA design.

Table S8. gRNA and their coverage for cross-species *ADR1* gRNA design.

Table S9. gRNA and their coverage for cross-species *ADR1* gRNA design for multi-PAM xCas9 (3' NG, NNG, GAA, GAT).

Table S10. gRNA and their coverage for cross-species *NRG1.1* gRNA design.

Table S11. gRNA and their coverage for cross-species *NRG1.1* gRNA design for multi-PAM xCas9 (3' NG, NNG, GAA, GAT).

Table S12. Comparison table with similar gRNA design tools.

2 Supplementary Figures

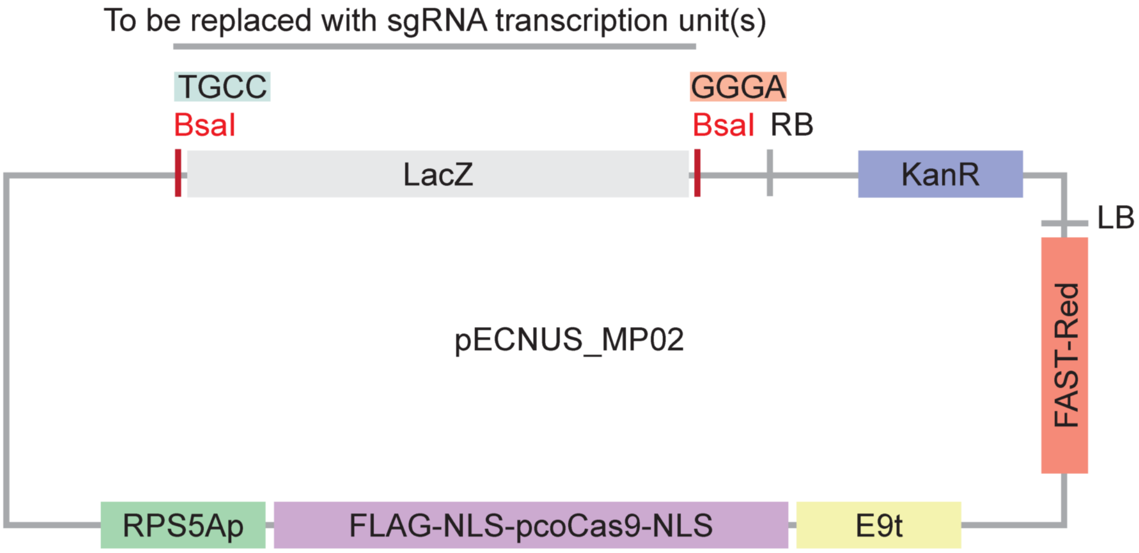

Figure S1. Schematic representation of binary acceptor vector pECNUS_MP02. (A) pECNUS_MP02 is an alternative to pECNUS_MP01 for the assembly of more than six gRNA cassettes. The LacZ selection marker in pECNUS_MP02 is flanked by two BsaI recognition sites, which replace the BpiI recognition sites in pECNUS_MP01. Apart from these two BsaI recognition sites, all other components and vector architecture are identical to pECNUS_MP01. To assemble more than six gRNA cassettes in a final binary vector, level M vectors, which use BsaI recognition sites for excision of tandemly inserted fragments in the final assembly, are required as intermediate cloning vectors to introduce an additional layer of multiplexing. pECNUS_MP02 was designed as an alternative to pECNUS_MP01 for compatibility with level M vectors. See also Figure S2 for detailed cloning process when seven gRNAs need to be assembled. Addgene numbers: 194343 (pECNUS_P1), 194344 (pECNUS_P2), 194345 (pECNUS_P3), 194346 (pECNUS_P4), 194347 (pECNUS_P5), 194348 (pECNUS_P6), 194349 (pECNUS_P7), 194350 (pECNUS_MP01), 194351 (pECNUS_MP02).

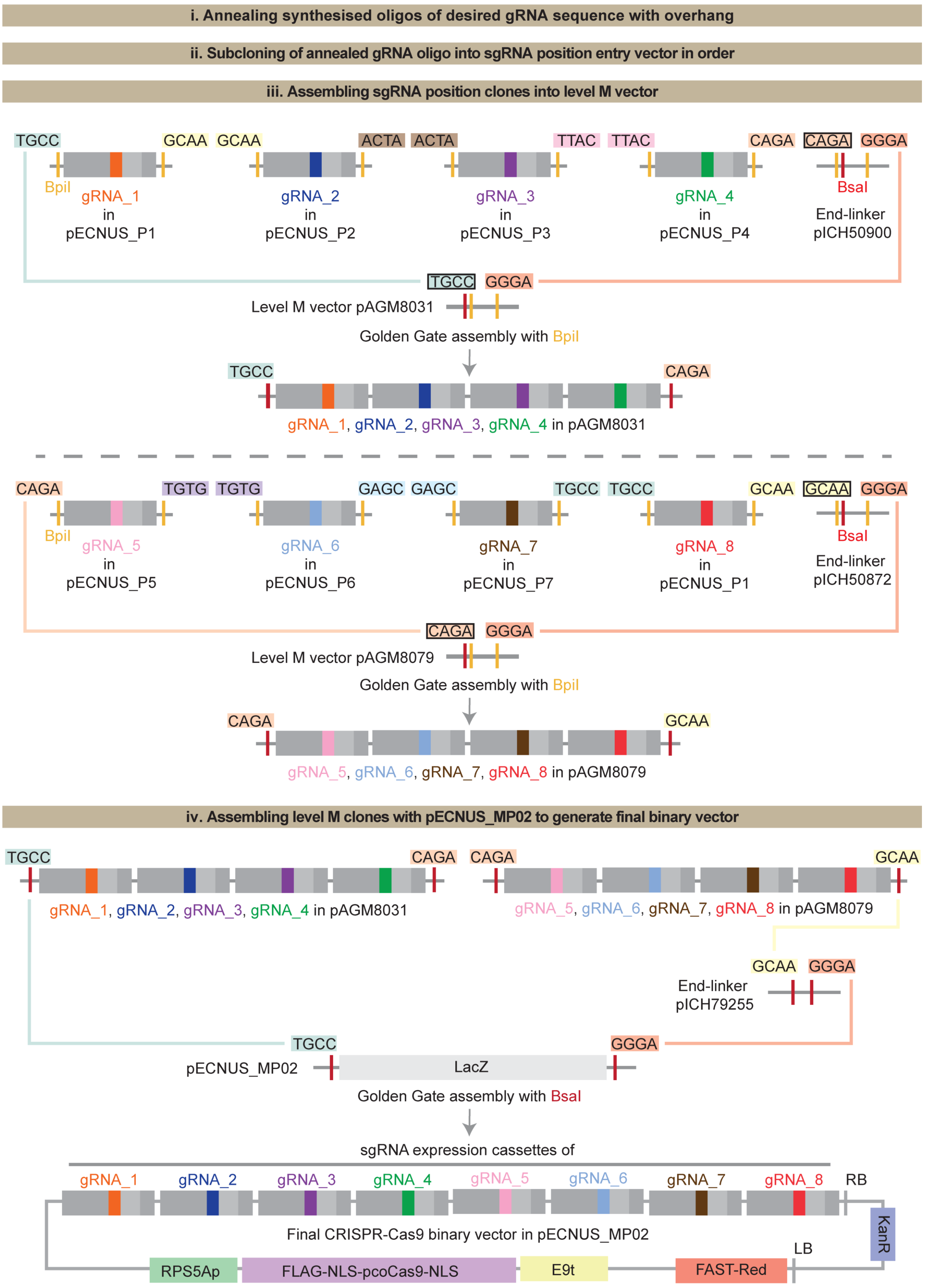
i. Annealing synthesised oligos of desired gRNA sequence with overhang
ii. Subcloning of annealed gRNA oligo into sgRNA position entry vector in order
iii. Assembling sgRNA position clones into level M vector
TGCC
GCAA
GCAA
ACTA
ACTA
TTAC
TTAC
CAGA
CAGA
GGGA
BpiI
gRNA_1 in pECNUS_P1
gRNA_2 in pECNUS_P2
gRNA_3 in pECNUS_P3
gRNA_4 in pECNUS_P4
BsaI
End-linker pICH50900
TGCC
GGGA
Level M vector pAGM8031
Golden Gate assembly with BpiI
TGCC
CAGA
gRNA_1, gRNA_2, gRNA_3, gRNA_4 in pAGM8031
CAGA
TGTG
TGTG
GAGC
GAGC
TGCC
TGCC
GCAA
GCAA
GGGA
BpiI
gRNA_5 in pECNUS_P5
gRNA_6 in pECNUS_P6
gRNA_7 in pECNUS_P7
gRNA_8 in pECNUS_P1
BsaI
End-linker pICH50872
CAGA
GGGA
Level M vector pAGM8079
Golden Gate assembly with BpiI
CAGA
GCAA
gRNA_5, gRNA_6, gRNA_7, gRNA_8 in pAGM8079
iv. Assembling level M clones with pECNUS_MP02 to generate final binary vector
TGCC
CAGA
CAGA
GCAA
gRNA_1, gRNA_2, gRNA_3, gRNA_4 in pAGM8031
gRNA_5, gRNA_6, gRNA_7, gRNA_8 in pAGM8079
GCAA
GGGA
End-linker pICH79255
TGCC
GGGA
pECNUS_MP02
LacZ
Golden Gate assembly with BsaI
sgRNA expression cassettes of
gRNA_1
gRNA_2
gRNA_3
gRNA_4
gRNA_5
gRNA_6
gRNA_7
gRNA_8
RB
KanR
Final CRISPR-Cas9 binary vector in pECNUS_MP02
LB
RPS5Ap
FLAG-NLS-pcoCas9-NLS
E9t
FAST-Red

Figure S2. Schematic workflow of the sample assembly of eight sgRNA expression cassettes into a final binary vector (pECNUS_MP02) using the CRISPR-Cas9 multiplexing vector toolkit. The first two steps are identical to Figure S3. (i, ii) gRNA_1 to gRNA_7 can be subcloned into pECNUS_P1 to pECNUS_P7 in order; gRNA_8 should be inserted to pECNUS_P1. (iii) Next, position entry clones for gRNA_1 to gRNA_4 are mixed with end-linker pICH50900 and inserted into level M vector pAGM8031; position clones for gRNA_5 to gRNA_8 are mixed with end-linker pICH50872 to insert into level M vector pAGM8079. Both cloning reactions are performed via BpiI Golden Gate assembly. (iv) Finally, the two newly cloned level M vectors are mixed with end-linker pICH79255 to replace the LacZ component in pECNUS_MP02 by BsaI Golden Gate assembly to generate the final CRISPR binary vector that can express eight sgRNA cassettes along with a Cas9 expression cassette. While each level M vector only has a single BsaI recognition site, the end-linkers used to bridge the last position clone with the level M vectors also have a BsaI recognition site (iii), bringing the total number of BsaI recognition sites in an assemble level M clone to two. Within each end-linker, the first BpiI recognition site and the sole BsaI recognition site generate the same overhangs (outlined in black). Within each level M vector, the sole BsaI recognition site and the first BpiI recognition site also generate the same overhang (outlined in black). These overhangs are regenerated by BsaI digestion for the final assembly of level M clones into the binary vector. Addgene numbers: 194343 (pECNUS_P1), 194344 (pECNUS_P2), 194345 (pECNUS_P3), 194346 (pECNUS_P4), 194347 (pECNUS_P5), 194348 (pECNUS_P6), 194349 (pECNUS_P7), 194350 (pECNUS_MP01), 194351 (pECNUS_MP02).

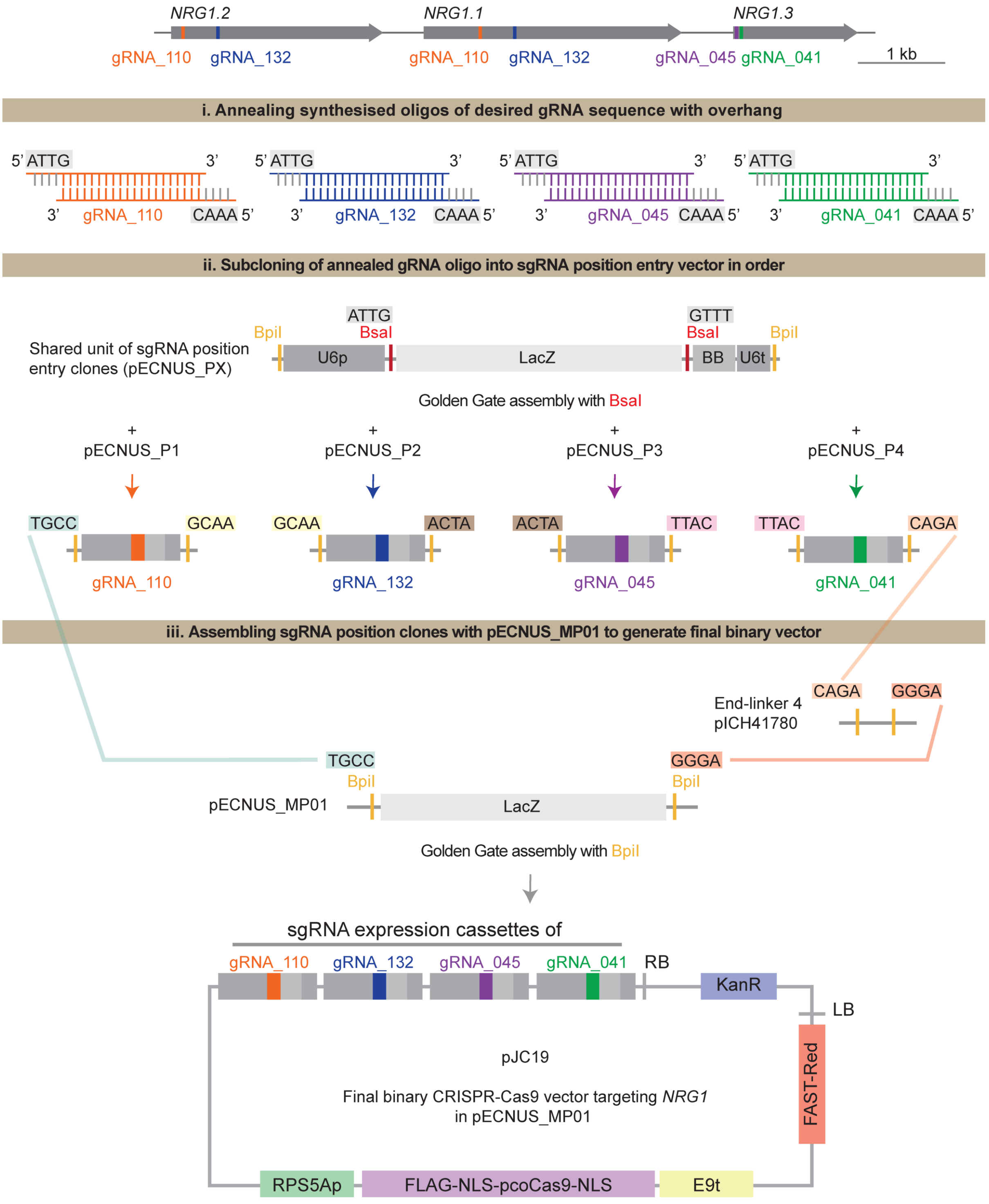
NRG1.2
NRG1.1
NRG1.3
gRNA_110
gRNA_132
gRNA_110
gRNA_132
gRNA_045
gRNA_041
1 kb
i. Annealing synthesised oligos of desired gRNA sequence with overhang
5' ATTG
3'
3'
gRNA_110
CAAA 5'
gRNA_132
gRNA_045
gRNA_041
ii. Subcloning of annealed gRNA oligo into sgRNA position entry vector in order
Shared unit of sgRNA position entry clones (pECNUS_PX)
BpiI
ATTG
BsaI
U6p
LacZ
GTTT
BsaI
BB
U6t
BpiI
Golden Gate assembly with BsaI
+
pECNUS_P1
pECNUS_P2
pECNUS_P3
pECNUS_P4
TGCC
GCAA
ACTA
TTAC
CAGA
gRNA_110
gRNA_132
gRNA_045
gRNA_041
iii. Assembling sgRNA position clones with pECNUS_MP01 to generate final binary vector
End-linker 4
pICH41780
CAGA
GGGA
TGCC
BpiI
GGGA
BpiI
pECNUS_MP01
LacZ
Golden Gate assembly with BpiI
sgRNA expression cassettes of
gRNA_110
gRNA_132
gRNA_045
gRNA_041
RB
KanR
LB
FAST-Red
pJC19
Final binary CRISPR-Cas9 vector targeting NRG1
in pECNUS_MP01
RPS5Ap
FLAG-NLS-pcoCas9-NLS
E9t

Figure S3. Schematic workflow of the assembly of gRNA cassettes into a final binary vector (pECNUS_MP01) for *NRG1.1/2/3* knockout using the CRISPR-Cas9 multiplexing vector toolkit. Forward and reverse DNA oligos of desired gRNA sequence were synthesized with added 5' ATTG 3' and 5' AAAC 3' overhangs respectively. (i) Paired forward and reverse oligos were annealed to generate sticky ends. (ii) Next, sgRNA expression cassettes were generated by separately subcloning the annealed oligos for different gRNA into sgRNA position entry vectors via Golden Gate assembly with BsaI: gRNA_110 were inserted into pECNUS_P1, gRNA_132 into pECNUS_P2, gRNA_045 into pECNUS_P3, and gRNA_041 into pECNUS_P4. Blue/white selection and Sanger sequencing were conducted to identify correctly cloned vectors. (iii) Finally, the four cloned sgRNA position vectors with inserted gRNA (from pECNUS_P1 to pECNUS_P4), along with the fourth end-linker pICH41780 and acceptor vector pECNUS_MP01, were mixed in a single reaction for another round of Golden Gate assembly using BpiI to generate pJC19. Blue/white selection and Sanger sequencing were once again performed to confirm correct assembly of the final binary vector pJC19. Addgene numbers: 194343 (pECNUS_P1), 194344 (pECNUS_P2), 194345 (pECNUS_P3), 194346 (pECNUS_P4), 194347 (pECNUS_P5), 194348 (pECNUS_P6), 194349 (pECNUS_P7), 194350 (pECNUS_MP01), 194351 (pECNUS_MP02).

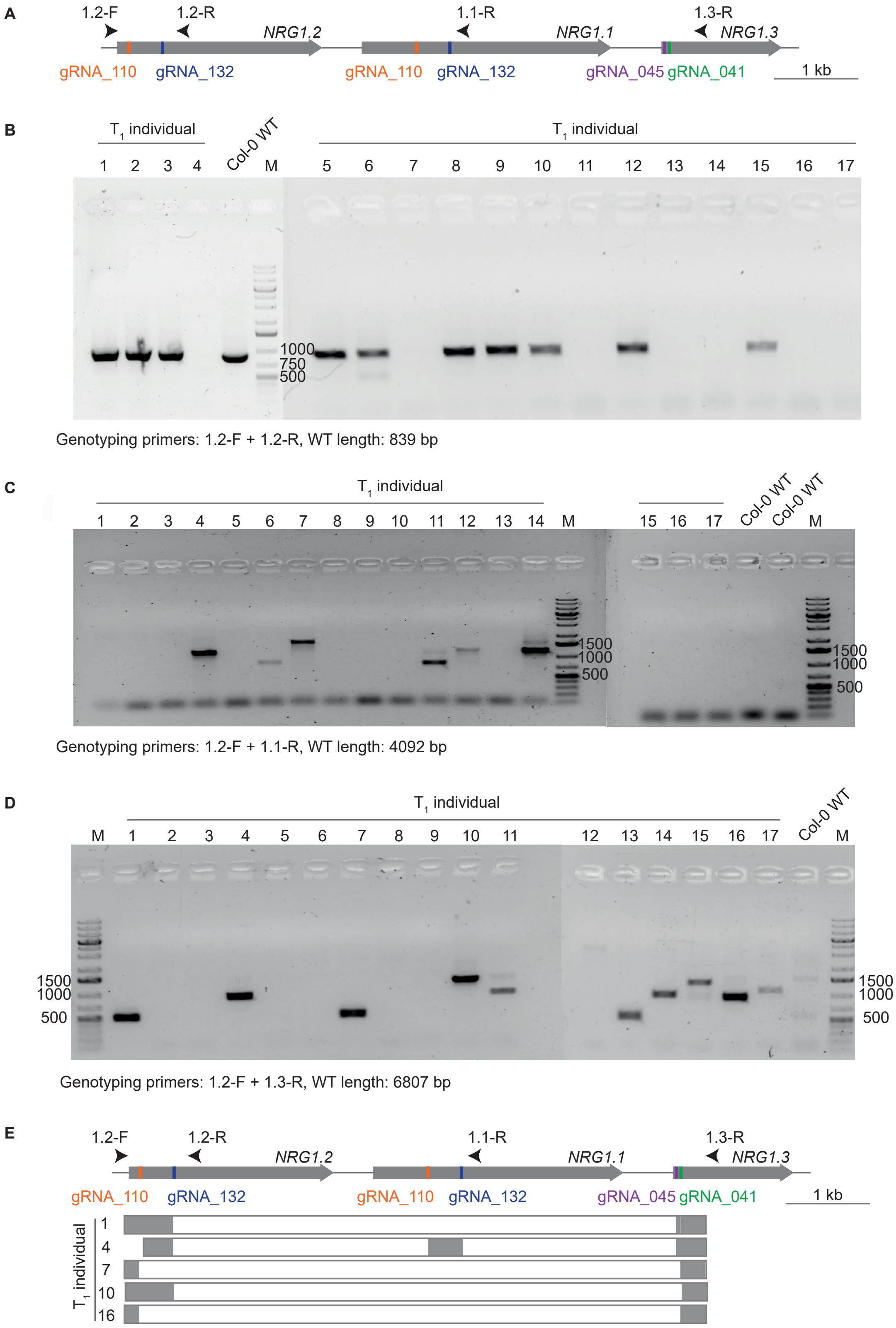
A
1.2-F
1.2-R
NRG1.2
1.1-R
NRG1.1
1.3-R
NRG1.3
gRNA_110
gRNA_132
gRNA_045
gRNA_041
1 kb
B
T1 individual
Col-0 WT
M
1000
750
500
Genotyping primers: 1.2-F + 1.2-R, WT length: 839 bp
C
T1 individual
1500
1000
500
Genotyping primers: 1.2-F + 1.1-R, WT length: 4092 bp
D
T1 individual
Col-0 WT
M
1500
1000
500
Genotyping primers: 1.2-F + 1.3-R, WT length: 6807 bp
E
1.2-F
1.2-R
NRG1.2
1.1-R
NRG1.1
1.3-R
NRG1.3
gRNA_110
gRNA_132
gRNA_045
gRNA_041
1 kb
T1 individual
1
4
7
10
16

Figure S4. Large fragment deletion was detected in multiple T_1 individuals transformed with pJC19 for simultaneous knockout of *NRG1.1/2/3*. **(A)** Schematic representation of the genomic arrangement of *NRG1.1/2/3*, with the positions of gRNAs and PCR genotyping primers (arrowheads) indicated. Primer names were abbreviated as 1.2-F (NRG1.1_110_132-F), 1.2-R (NRG1.2_110_132-R), 1.1-R (NRG1.3_110_132-R), and 1.3-R (NRG1.3-R). **(B-D)** PCR genotyping results using primer 1-2-F paired with primers 1.2-R **(B)**, 1.1-R **(C)**, and 1.3-R **(D)** for T_1 individuals transformed with pJC19, with wildtype (WT) Col-0 as control. **(E)** Alignment of Sanger sequencing results of PCR products of selected T_1 individuals (1, 4, 7, 10, and 16) from **(D)** with the wildtype genomic arrangement of *NRG1.1/2/3* confirming large deletions between gRNA editing sites. Regions in grey and white indicate successful alignment and gaps respectively between sequencing results and WT sequence.

3 Supplementary Methods

3.1 CRISPR-Cas9 vector pKI-1.1R subcloning protocol

pKI-1.1R has low transformation efficiency but following this protocol faithfully will yield 95% success rate. Deviation from protocol can easily yield you zero transformation.

3.1.1 sgRNA design and planting

Forward oligo: 5' – ATTGN$_{20}$ or ATTGN$_{19}$* – 3'
Reverse oligo: 3' – N$_{20}$CAAA or N$_{19}$CAAA*
*N$_{19}$ is used whenever the first base of 20-base gRNA starts with "G"

gRNA design should be done with Rachelle Lee's MINORg whenever possible.

Design NGS primers after gRNA design to ensure targeted site can be sequenced. Test NGS primers beforehand if possible.

Design two gRNA per gene target, in case one gRNA does not work. Ensure the two gRNAs are spaced as far apart as possible to [1] tackle potential alternative start sites and [2] avoid potential heterochromatin on one site.

The subcloning process should take less than two weeks (1st week: subclone into E. coli; 2nd week: transform into Agrobacteria). Thus you can plant your Arabidopsis as soon as you start cloning.

3.1.2 Anneal gRNA oligos

Reagents

Reagent	Volume (µL)
Forward oligo (100 µM stock)	1
Reverse oligo (100 µM stock)	1
NEB T4 Ligase buffer	1
Water	7

Cycle

Temperature	Time (min)
37℃	30
95℃	5
↓ cool down	5℃/min
25℃	5
4℃	∞ overnight

3.1.3 AarI digestion of pKI series and dephosphorylation

The following reagent mixture yields sufficient digested pKI-1.1R for 4 ligation reactions:

Reagent	Volume (µL)
10X AaRI Buffer	2
50x oligo	0.2
pKI-1.1R (1 µg)	X
AaRI	0.5
Water	Top up to 50

Incubate at 37°C overnight for not more than 16 hours (to prevent star activity). Heat inactivate at 65°C for 20 minutes.

Load sample with DNA loading dye. Run gel at 120V for 30 minutes with 1 Kb Plus ladder. Gel extract 18.5 kB band. Follow manufacturer's protocol but before elution, dry column on 55 ℃ for 5 minutes. Elute with 50 µL elution buffer.

3.1.4 Ligation, transformation and selection

Ligation reagents

Reagent	Volume (µL)
Digested pKI-1.1R	10*
NEB Ligase buffer	2
NEG Ligase	0.5
10 mM ATP	25
Annealed oligos (diluted 250-fold)	1
Water	4.5

*10 µL digested pKI-1.1R is critical to overcome low transformation efficiency of this vector.

Ligate overnight at 4°C. Allow further ligation at 22°C (RTP) for 20 minutes. Heat inactivate at 65°C for 10 minutes.

Chemical transformation with XL-blue cells (XL-blue is made with tetracyclin and are thus contaminant-free; one colony can be picked from the plate and miniprep'ed without colony PCR. Successful subcloning is near 100% if this protocol is replicated faithfully). Plate on spectinomycin plate.

Pick one colony (no need for colony PCR). Miniprep and sequence with "U6 promoter sgRNA sequencing_2" (5'-AAG CAG GCC CAT TTA TAT G-3').

Transform subcloned pKI-1.1R into GV3101 Agrobacteria.
